# Rapid and Efficient Purification of Low-Concentration Fluoride-Containing Water Using Cationic Chitosan Fibers

**DOI:** 10.3390/gels12030195

**Published:** 2026-02-26

**Authors:** Zhe Liu, Dongfang Wang, Yan Zhu, Songlin Wang

**Affiliations:** 1 School of Environmental Science and Engineering, Huazhong University of Science and Technology, Wuhan 430074, China; 2 Hubei Provincial Academy of Eco-Environmental Sciences (Provincial Ecological Environment Engineering Assessment Center), Wuhan 430072, China; 3 Hubei Key Laboratory of Pollution Damage Assessment and Environmental Health Risk Prevention and Control, Wuhan 430072, China

**Keywords:** fluoride, adsorption, chitosan fibers, low-concentration, ion exchange

## Abstract

In this study, a bio-based adsorbent, quaternary ammonium-modified cationic chitosan fibers (CCFs), were developed and systematically evaluated for fluoride removal from low-concentration aqueous medium, with particular emphasis on adsorption performance, regeneration behavior, practical applicability, and adsorption mechanisms. The results demonstrate that the fluoride adsorption capacity of CCFs is approximately 15.8 times higher than that of unmodified chitosan fibers (CFs). Furthermore, CCFs exhibit superior fluoride adsorption capacity and remarkably rapid kinetics relative to previously reported chitosan-based adsorbents in the literature. The adsorption process fits well with the pseudo-second-order kinetic model and reaches equilibrium within 10 min. Adsorption isotherm data are well described by both Langmuir and Freundlich models, with a maximum adsorption capacity of 28.5 mg/g. CCFs also show excellent regeneration performance, achieving efficient fluoride desorption within 3 min using a 0.02 mol/L NaCl solution, with no noticeable loss in adsorption capacity after five consecutive adsorption–desorption cycles. The adsorption performance remains effective in natural surface water containing competing ions. Mechanistic investigations reveal that fluoride adsorption is dominated by electrostatic attraction between quaternary ammonium groups (R_4_N^+^) on the CCFs surface and fluoride ions, accompanied by ion exchange with chloride ions. Owing to their high efficiency, rapid kinetics, metal-free nature, and facile regeneration, the CCFs developed in this study represent a promising bio-based adsorbent for the advanced purification of low-concentration fluoride-containing water.

## 1. Introduction

Fluoride contamination in water is a globally recognized environmental and public health concern, primarily arising from the dissolution and complexation of fluoride-bearing minerals in ores and aquifers. This phenomenon has caused widespread drinking water safety issues in countries such as India, Pakistan, China, and Germany [1]. Fluoride is an essential trace element for human health, as appropriate intake contributes to bone strengthening and the prevention of dental caries. However, long-term exposure to excessive fluoride can lead to serious health problems, including dental fluorosis, skeletal fluorosis, and neurological disorders. Consequently, stringent regulations have been established worldwide to control fluoride levels in drinking water. The World Health Organization (WHO) recommends a maximum fluoride concentration of 1.5 mg/L, while China has adopted a stricter limit of 1.0 mg/L [2]. Industrial activities such as coal combustion, ceramic manufacturing, phosphorus chemical production, and metal smelting discharge substantial amounts of fluoride into aquatic environments, representing the primary sources of inorganic fluoride pollution [3]. In recent years, the rapid expansion of strategic emerging industries, including photovoltaic power generation, semiconductor manufacturing, and lithium-ion battery production, has led to a continuous increase in fluoride-containing wastewater discharges, further intensifying the risks posed by inorganic fluoride to water environmental safety and public health [4]. Therefore, the development of efficient, cost-effective, and environmentally friendly technologies for deep fluoride removal is of critical importance.

A variety of treatment technologies have been applied for fluoride removal from water, including chemical precipitation [5], coagulation [6], membrane separation [7], ion exchange [8], and adsorption [9]. Among these, the removal of low-concentration fluoride remains particularly challenging due to the small ionic radius and high electronegativity of fluoride ions (F^−^). For example, the calcium hydroxide precipitation method, which is widely used in industry, is effective for high-fluoride wastewater but exhibits significantly reduced efficiency when fluoride concentrations fall below 8–10 mg/L. In addition, this method generates large quantities of fluoride-containing sludge, resulting in high disposal costs and operational challenges [10]. In contrast, adsorption has emerged as a promising approach for low-concentration fluoride removal owing to its operational simplicity, high efficiency, and low risk of secondary pollution [11].

To date, extensive research has been conducted on adsorbent materials for fluoride removal, including metal-based materials, natural minerals, carbon-based materials, and nanomaterials [12]. Metal-based adsorbents, such as aluminum-based and rare-earth-based materials, often exhibit high fluoride adsorption capacities but suffer from drawbacks including high cost, narrow pH applicability, and potential metal ion leaching [2,13]. Natural minerals, such as zeolites and clays, are abundant and inexpensive; however, their adsorption capacities and selectivity for fluoride are generally limited [14]. Carbon-based materials possess large specific surface areas, but effective fluoride adsorption typically requires surface modification, such as metal oxide loading [15]. Although emerging nanomaterial adsorbents show excellent performance, their large-scale application is hindered by difficulties in solid–liquid separation, potential secondary pollution, and high production costs [16]. Overall, most existing fluoride adsorbents suffer from one or more limitations, including slow adsorption kinetics, insufficient performance at low fluoride concentrations, strong pH dependence, or poor regenerability.

In recent years, bio-based adsorbents have attracted increasing attention due to their renewability, low toxicity, and ease of functionalization. Chitosan, the second most abundant natural polysaccharide, exhibits excellent biocompatibility, biodegradability, abundant functional groups (e.g., –NH_2_ and –OH), and high modifiability, making it a promising candidate for environmental remediation applications. Previous studies have demonstrated that metal-modified chitosan materials can achieve effective fluoride removal; however, these systems often suffer from narrow pH range, susceptibility to interference from coexisting ions, and secondary pollution caused by metal leaching [17].

Based on our previous studies, chitosan fibers modified with quaternary ammonium groups have exhibited outstanding adsorption performance for various anionic pollutants, including diclofenac and gold–cyanide complexes, characterized by exceptionally high adsorption capacities and rapid kinetics [18,19]. Moreover, the fibrous morphology provides distinct advantages in solid–liquid separation and regeneration. These features suggest that quaternary ammonium-functionalized chitosan fibers, possessing abundant surface cationic sites, may serve as highly efficient adsorbents for fluoride removal. Accordingly, this study aims to systematically investigate the adsorption performance and mechanisms of cationic chitosan fibers (CCFs) for fluoride ions in aqueous systems, with a particular focus on low-concentration fluoride removal under complex ionic conditions. The findings are expected to provide both a material basis and mechanistic insights for the development of advanced fluoride removal technologies applicable to industrial wastewater treatment, drinking water safety, and groundwater remediation.

## 2. Results and Discussion

### 2.1. Adsorption Studies

#### 2.1.1. Single-Point Assessment for Fluoride Adsorption

Chitosan possesses abundant amino and hydroxyl groups on its surface, which enable fluoride adsorption through hydrogen bonding or electrostatic interactions with protonated amino groups [20]. However, the fluoride adsorption capacity of pristine chitosan fibers (CFs) was very limited, reaching only 1.6 mg/g (Figure S1). In contrast, under the same conditions, cationic chitosan fibers (CCFs) prepared by quaternization modification exhibited a significantly enhanced fluoride adsorption capacity of 25.3 mg/g, approximately 15.8 times higher than that of CFs. The point of zero charge (pH_pzc_) of CCFs was found to be 10.40 [19], indicating a positively charged surface in the studied pH range. This remarkable improvement can be attributed to the introduction of abundant positively charged quaternary ammonium groups, which promote fluoride uptake through strong electrostatic attraction, thereby substantially increasing the number of effective adsorption sites.

#### 2.1.2. pH Effect

**Figure 1:**
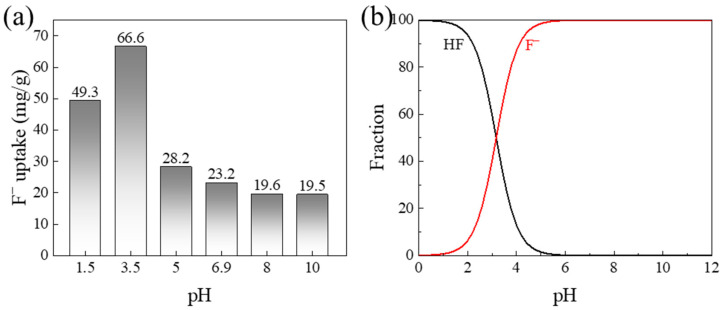
Adsorption performance of CCFs for fluoride: (**a**) effect of pH, and (**b**) fluoride species at different pH values.

Solution pH is a critical parameter governing fluoride adsorption, as it simultaneously affects the surface charge of the adsorbent and the speciation of fluoride ions in solution [21]. The effect of solution pH on the adsorption of fluoride by cationic chitosan fibers (CCFs) was investigated within the pH range of 1.5 to 10. As shown in Figure 1a, acidic conditions were favorable for adsorption, with an adsorption capacity of 49.3 mg/g at pH 1.5. As the pH increased to 3.5, the adsorption capacity increased to 66.6 mg/g. This enhancement is attributed to the gradual conversion of neutral HF species into negatively charged F^−^ ions with increasing pH (Figure 1b), which strengthens electrostatic interactions with the positively charged surface of CCFs. With further increases in pH, the adsorption capacity gradually decreased, primarily due to the reduction in surface positive charge density on CCFs under alkaline conditions, which weakens electrostatic attraction toward F^−^ [22].

Fluoride-containing wastewater (e.g., from semiconductor [5] and photovoltaic industries [23]) after pretreatment, as well as surface water used as drinking water sources, typically exhibits a near-neutral pH range (approximately 6–8). To better simulate practical application, subsequent adsorption kinetics and isotherm experiments in this study were conducted under the inherent pH (about 6.5) of the sodium fluoride solution.

#### 2.1.3. Adsorption Kinetics

**Figure 2:**
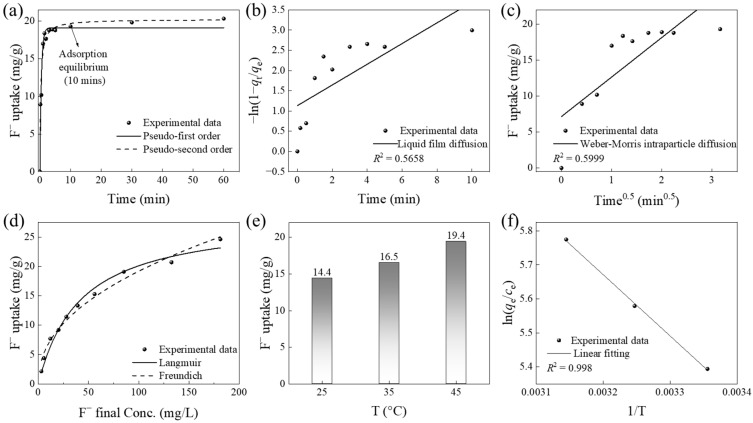
Adsorption performance of CCFs for fluoride: (**a**) adsorption kinetics, (**b**) liquid film diffusion model, (**c**) Weber–Morris intraparticle diffusion model, and (**d**) adsorption isotherms, (**e**) effect of temperature, and (**f**) absorption thermodynamic.

The adsorption kinetics of fluoride ions on CCFs under the conditions of an initial fluoride concentration of 100 mg/L, an adsorbent dosage of 1 g/L, and a temperature of 25 °C are shown in Figure 2a. A rapid adsorption behavior was observed, with approximately 83.7% of the equilibrium adsorption capacity (20.33 mg/g) achieved within the first minute. The uptake increased to 92.5% within 3 min, and the adsorption equilibrium was reached within approximately 10 min.

To further elucidate the kinetic characteristics, the pseudo-first-order kinetic (Equation (1)) and pseudo-second-order kinetic models (Equation (2)) were applied to the experimental data. The fitted parameters and correlation coefficients (*R*^2^) are presented in Table 1.

(1) $$q_{t}=q_{1}\left(1-\exp\left(-k_{1}t\right)\right)$$

(2) $$q_{t}=\frac{q_{2}^{2}k_{2}t}{1+q_{2}k_{2}t}$$

where *q_t_* represents the adsorption amount by CCFs at *t* times (mg/g). *k*_1_ (L/min) and *k*_2_ (g/(mg·min)) are the pseudo-first-order and pseudo-second-order adsorption rate constants. *q*_1_ (mg/g) and *q*_2_ (mg/g) are the equilibrium fluoride adsorption quantities calculated by the pseudo-first-order and pseudo-second-order models.

Although both models reasonably describe the adsorption process, the pseudo-second-order model provides a superior fit (*R*^2^ = 0.97) compared with the pseudo-first-order model (*R*^2^ = 0.95). In addition, the calculated equilibrium adsorption capacity (*q*_2_ = 20.24 mg/g) closely matches the experimental value (20.33 mg/g), indicating that fluoride adsorption onto CCFs is better described by the pseudo-second-order kinetic model. Further analysis using the liquid film diffusion (Equation (3)) and Weber–Morris intraparticle diffusion (Equation (4)) models (Figure 2b,c) shows that neither model yields a single linear relationship passing through the origin.

(3) $$-\ln\left(1-\frac{q_{t}}{q_{e}}\right)=k_{L}t+c_{L}$$

(4) $$q_{t}=k_{W-M}t^{\frac{1}{2}}+c_{W-M}$$

where *c_L_* and *c_W_*_−*M*_ denote the intercept terms for liquid film diffusion and the Weber–Morris intraparticle diffusion model, and *k_L_* (min^−1^) and *k_W_*_−*M*_ (mg/(g·min^1/2^) are the corresponding rate constants for liquid film diffusion and Weber–Morris intraparticle diffusion model, respectively.

The presence of multilinear behavior and non-zero intercepts indicates that fluoride adsorption onto CCFs is not solely controlled by physical diffusion. Rather, it is more likely governed by surface kinetic processes, primarily influenced by the interactions between the adsorbent’s active sites and the fluoride ions.

#### 2.1.4. Adsorption Isotherms

The adsorption isotherms of fluoride on CCFs at 25 °C, with an adsorbent dosage of 1 g/L, an initial fluoride concentration range of 5 to 200 mg/L, and at neutral pH, are shown in Figure 2d. At low equilibrium concentrations, the adsorption amount increases rapidly, indicating the presence of abundant high-affinity sites on the adsorbent surface. As concentration continues to increase, the curve gradually approaches a plateau, suggesting progressive saturation of available surface sites. The isotherm data were fitted using the Langmuir (Equation (5)) and Freundlich (Equation (6)) models [24], and the corresponding parameters are listed in Table 1.

(5) $$q_{e}=\frac{q_{max}bc_{e}}{1+bc_{e}}$$

(6) qe=kFce1n

where *c_e_* (mg/L) and *q_e_* (mg/g) are the equilibrium concentration and adsorption capacity, respectively. *b* (L/mg) and *q_max_* (mg/g) are the Langmuir constants and the maximum adsorption capacity; *k_F_* ((mg/g)/(L/mg)^1/*n*^) and *n* are Freundlich constants related to adsorption capacity and intensity.

Both models exhibit excellent fitting performance, with correlation coefficients (*R*^2^) of 0.98. To further compare the goodness of fit, the reduced chi-square (χ^2^_red_) reported by Origin’s nonlinear fitting was used. The Langmuir model gave a lower χ^2^_red_ (0.94) than the Freundlich model (1.04), indicating a slightly better fit for the Langmuir model. The Freundlich constant n (2.23 ± 0.13) exceeds 1, indicating favorable adsorption and strong affinity of CCFs for F^−^, particularly at low concentrations. The maximum adsorption capacity derived from the Langmuir model reaches 28.5 ± 1.4 mg/g, highlighting the strong fluoride uptake capability of CCFs. The comparable fitting quality of the two models suggests that the adsorption process may involve a composite mechanism, combining features of monolayer coverage and surface heterogeneity.

#### 2.1.5. Adsorption Thermodynamic

Solution temperature serves as a critical parameter influencing adsorption processes. Furthermore, given the inherent variability in industrial wastewater discharge temperatures and the seasonal and regional fluctuations of surface water, evaluating the impact of temperature on fluoride ion adsorption by CCFs in aqueous solutions is of substantial significance for both deepening the understanding of their adsorption behavior and guiding their practical applications.

Adsorption experiments of fluoride ions in aqueous solutions by CCFs were performed at 25, 35, and 45 °C. The resulting adsorption capacities are depicted in Figure 2e, clearly indicating that the adsorption of fluoride onto CCFs enhances with rising temperature, demonstrating that higher temperatures are favorable for the adsorption process.

To further investigate the thermodynamic nature of this adsorption process, the Gibbs free energy change (Δ*G*°), enthalpy change (Δ*H*°), and entropy change (Δ*S*°) were derived and computed using Equations (7) and (8) [25]. These thermodynamic parameters are summarized in Figure 2f and Table 1.

The negative Δ*G*° values indicate spontaneous adsorption. Furthermore, as temperature increases, the magnitude of −Δ*G*° gradually rises, suggesting that higher temperatures enhance the spontaneity of the adsorption process. Considering that Δ*H*° > 0 and Δ*S*° > 0, the adsorption process is primarily an entropy-driven phenomenon, resulting from the synergistic action of physisorption and weak chemisorption. The positive Δ*S*° value indicates that the adsorption process leads to an increase in the overall disorder of the system. This is primarily because the migration of adsorbate ions from the aqueous phase to the solid–liquid interface disrupts their hydration shells, releasing bound water molecules into the bulk solvent. Such release increases the degrees of freedom and configurational entropy of the solvent, resulting in a net positive change in total entropy [2].

(7) $${\Delta G}^{o}=-RT\ln\frac{q_{e}}{c_{e}}$$

(8) $$\ln\frac{q_{e}}{c_{e}}=\frac{\Delta S^{o}}{R}-\frac{\Delta H^{o}}{R}\times \frac{1}{T}$$

#### 2.1.6. Comparison of Adsorption Performance

A comparison between CCFs and previously reported chitosan-based fluoride adsorbents is summarized in Table 2. As shown, CCFs exhibit distinct comprehensive advantages in terms of both adsorption capacity and adsorption kinetics. Under mildly acidic conditions (pH = 3.5), the fluoride adsorption capacity of CCFs reaches 66.6 mg/g. Under near-neutral conditions, the maximum adsorption capacity (*q_max_* = 28.5 mg/g) is comparable to that of high-performance chitosan–metal composite adsorbents, such as zirconium/lanthanum co-modified chitosan/polyvinyl alcohol (30.73 mg/g) [26], and significantly exceeds that of most single-metal-modified chitosan materials, which typically exhibit adsorption capacities in the range of 8–20 mg/g. Notably, the high adsorption performance of CCFs is achieved through non-metallic quaternary ammonium functionalization, fundamentally eliminating the risk of secondary pollution associated with metal ion leaching. This characteristic represents a critical advantage over metal-based chitosan adsorbents, particularly for applications involving drinking water treatment.

In terms of adsorption kinetics, CCFs demonstrate an even more pronounced advantage. Fluoride adsorption reaches equilibrium within approximately 10 min, which is substantially faster than that of most reported chitosan-based adsorbents, for which equilibrium times typically range from 30 to 480 min. The pseudo-second-order rate constant (*k*_2_ = 0.18 g/(mg·min)) ranks among the highest values reported for similar materials, while the pseudo-first-order rate constant (*k*_1_ = 2.09 L/min) is markedly higher than those of the comparative adsorbents listed in Table 2, confirming the exceptionally rapid initial adsorption rate of CCFs.

Overall, CCFs integrate high adsorption capacity, ultrafast adsorption kinetics, and metal-free environmental safety. Compared with conventional modification strategies that rely heavily on metal loading or complex material architectures, the present fiber-based cationic modification strategy offers a simple, efficient, and environmentally benign pathway for the development of next-generation bio-based defluoridation adsorbents.

#### 2.1.7. Regeneration and Reusability

**Figure 3:**
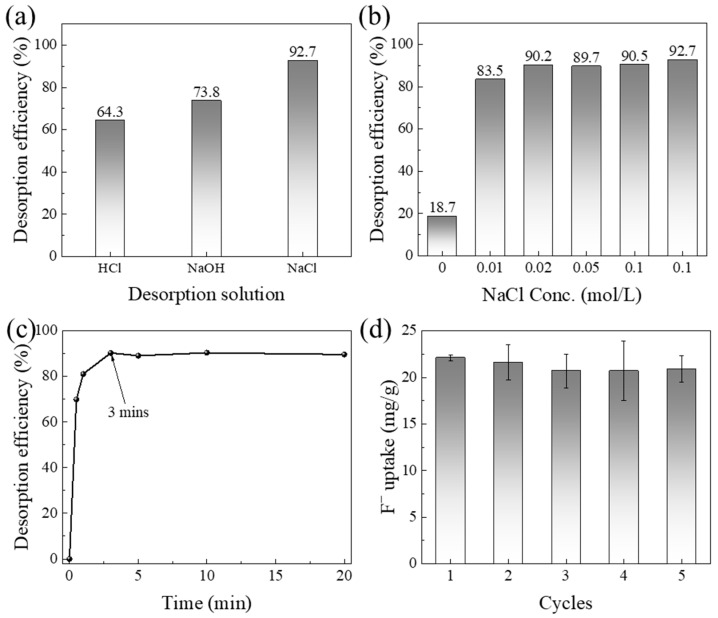
Evaluation of the desorption performance of CCFs: (**a**) regenerant screening, (**b**) optimization of NaCl concentration, (**c**) desorption kinetics, and (**d**) adsorption–desorption cycling.

Regeneration and reusability are critical indicators for evaluating the economic feasibility and practical applicability of adsorbents. The regeneration performance of CCFs was systematically investigated through regenerant screening, regenerant concentration optimization, desorption kinetics, and cyclic adsorption–desorption experiments, as shown in Figure 3a–d. Initially, fluoride-loaded CCFs were treated with 0.50 mol/L HCl, NaOH, and NaCl solutions to evaluate desorption efficiency. As shown in Figure 3a, NaCl solution exhibited the highest desorption efficiency, achieving a fluoride desorption rate of 92.7%. Further optimization of NaCl concentration (Figure 3b) revealed that increasing the NaCl concentration from 0 to 0.02 mol/L resulted in a sharp increase in desorption efficiency from 22.4% to 90.2%. A further increase to 0.50 mol/L produced only a marginal improvement. Therefore, 0.02 mol/L NaCl was selected as the optimal regenerant for subsequent experiments.

Desorption kinetics using 0.02 mol/L NaCl (Figure 3c) show that approximately 81.1% of the adsorbed fluoride was released within the first minute, and over 90% desorption was achieved within 3 min, indicating rapid and efficient regeneration. After five consecutive adsorption–desorption cycles (Figure 3d), no significant decrease in fluoride adsorption capacity was observed, demonstrating excellent cyclic stability of CCFs. These results indicate that CCFs possess not only high adsorption capacity but also fast regeneration kinetics and robust reusability, which are essential for sustainable water treatment applications.

#### 2.1.8. Fluoride Adsorption from Low-Concentration Real Water

**Figure 4:**
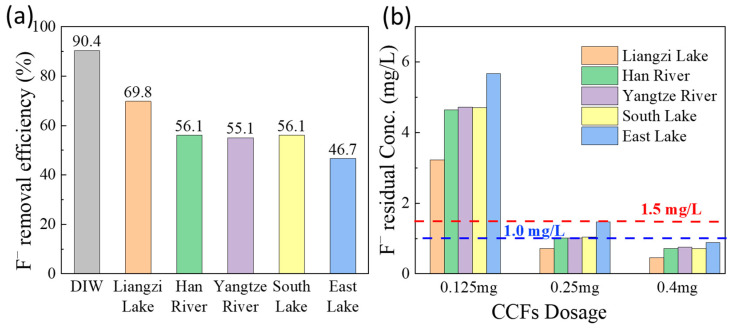
Adsorption performance in low-fluoride real surface water: (**a**) fluoride removal efficiency at an adsorbent dosage of 0.01 g, and (**b**) effect of adsorbent dosage.

High-fluoride wastewater is typically treated using chemical precipitation or flocculation, whereas adsorption is more suitable for the removal of low-concentration fluoride [10]. To assess the performance of CCFs under realistic conditions, five surface water samples collected from the Yangtze River, Han River, East Lake, South Lake, and Liangzi Lake were spiked to an initial fluoride concentration of 10 mg/L and subjected to adsorption experiments. The sampling locations, background anion concentrations, and total organic carbon (TOC) levels are summarized in Table 3, and the corresponding adsorption results are shown in Figure 4a.

At a fixed adsorbent dosage of 0.01 g, fluoride removal efficiencies in the surface water samples ranged from 46.7% to 69.8%, which were consistently lower than that observed in deionized water (90.4%). This reduction can be attributed to the presence of coexisting inorganic anions and dissolved organic matter in natural waters, which compete with fluoride ions for adsorption sites on the CCFs surface. Analysis of anion compositions (Table 3) reveals a clear correlation between fluoride removal efficiency and the concentrations of competing anions. Liangzi Lake, which contained the lowest levels of Cl^−^, HCO_3_^−^, and SO_4_^2−^, exhibited the highest fluoride removal efficiency (69.8%), whereas East Lake, with the highest concentrations of these anions, showed the lowest removal efficiency (46.7%). Although South Lake contained lower HCO_3_^−^ concentrations than the Yangtze and Han rivers, its relatively higher Cl^−^ and SO_4_^2−^ levels resulted in comparable fluoride removal efficiencies. Increasing the dosage of CCFs (Figure 4b) led to a gradual decrease in residual fluoride concentration in all surface water samples. At a dosage of 0.25 g per 10 mL, the effluent fluoride concentration in all samples met the WHO drinking water standard (<1.5 mg/L). Further increasing the dosage to 0.4 g reduced the fluoride concentration below 1.0 mg/L, satisfying the Chinese drinking water standard.

### 2.2. Characteristics of CCFs and Adsorption Mechanisms Studies

#### 2.2.1. Morphology

The morphology of original and fluoride-loaded CCFs was examined by scanning electron microscopy (SEM), as shown in Figure S2. The CCFs exhibit irregular fibrous morphologies, with individual fibers appearing ribbon-like or helically coiled and diameters ranging from approximately 50 to 200 μm. The fiber surfaces are continuous and intact, displaying slight undulations and micro-scale roughness generated during the spinning and modification processes. No significant fiber agglomeration or structural collapse is observed. After fluoride adsorption, no noticeable changes in fiber morphology, diameter, or surface texture are detected. The structural integrity of the fibers remains well preserved, indicating that fluoride adsorption does not disrupt the overall fiber structure. This morphological stability is beneficial for repeated adsorption–desorption cycles and practical water treatment applications.

#### 2.2.2. DFT Calculations

**Figure 5:**
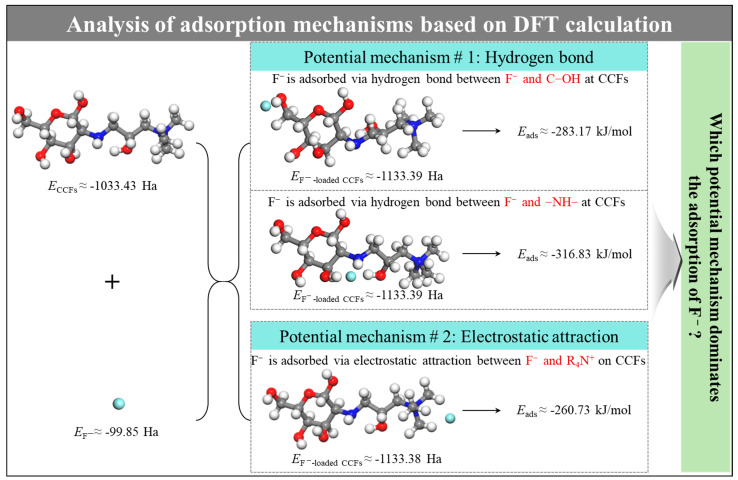
DFT calculated adsorption energies (Eads) of F^−^ on CCFs.

Based on the molecular structure of CCFs and the electronic characteristics of fluoride ions, two primary interaction pathways may contribute to fluoride adsorption: (i) hydrogen bonding between fluoride ions and hydroxyl or amino groups on the chitosan backbone, and (ii) electrostatic interactions between fluoride ions and positively charged quaternary ammonium groups on the fiber surface. To evaluate the relative thermodynamic favorability of these interactions, density functional theory (DFT) calculations were conducted to determine the adsorption energies of fluoride ions at different active sites, as illustrated in Figure 5.

The calculated adsorption energies indicate that fluoride exhibits the most negative adsorption energy when interacting with amino groups (−316.83 kJ/mol), followed by hydroxyl groups (−283.17 kJ/mol), while interaction with quaternary ammonium groups results in a comparatively less negative adsorption energy (−260.73 kJ/mol). From a thermodynamic perspective, more negative adsorption energies correspond to more energetically stable adsorption configurations, suggesting that hydrogen bonding interactions with amino or hydroxyl groups are thermodynamically favorable under the idealized computational conditions.

However, it should be emphasized that DFT calculations represent isolated and idealized interaction scenarios, which do not fully account for competitive solvation effects, ion hydration, and site accessibility in aqueous systems. Therefore, while hydrogen bonding interactions may be energetically favorable at the molecular level, their effective contribution to fluoride adsorption under experimental conditions must be evaluated in conjunction with experimental evidence.

#### 2.2.3. FTIR

**Figure 6:**
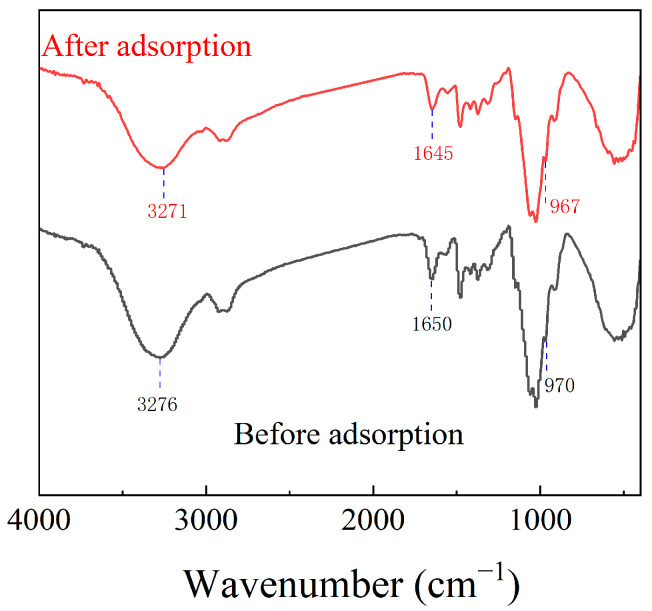
FTIR of CCFs before and after fluoride adsorption.

Fourier transform infrared (FTIR) spectroscopy was employed to investigate changes in surface functional groups of CCFs induced by fluoride adsorption. The corresponding spectra are presented in Figure 6, and characteristic absorption bands are summarized in Table S1 [42]. After fluoride adsorption, minor spectral changes were observed. Specifically, the stretching vibration bands associated with hydroxyl and amino groups shifted slightly from 3276 to 3271 cm^−1^ and from 1650 to 1645 cm^−1^, respectively, accompanied by a slight decrease in peak intensity. In addition, the characteristic vibration band of quaternary ammonium groups (R_4_N^+^) exhibited a marginal shift from 970 to 967 cm^−1^.

These subtle peak shifts indicate local perturbations in the chemical environment of the corresponding functional groups following fluoride adsorption. However, the magnitude of these changes is insufficient to independently quantify the relative contribution of hydrogen bonding versus electrostatic interactions. Therefore, FTIR results alone cannot conclusively establish the dominant adsorption mechanism and must be interpreted in combination with other analytical techniques.

The slight shifts in these peak positions within the FTIR spectrum are insufficient to independently confirm the role of hydroxyl/amino groups in the adsorption process or the precise contribution of quaternary ammonium groups [27,43,44]. Comprehensive verification requires integration with other characterization methods.

#### 2.2.4. XPS

**Figure 7:**
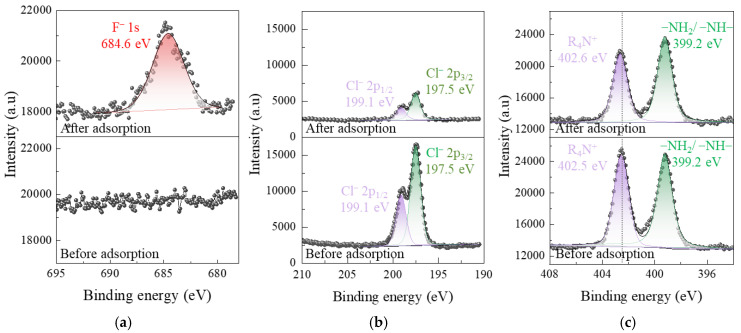
XPS spectra of CCFs before and after fluoride adsorption: (**a**) F 1s, (**b**) Cl 2p, and (**c**) N 1s spectra.

To further clarify the adsorption mechanism, XPS analysis was performed on CCFs before and after fluoride adsorption (Figure 7). After fluoride adsorption, a distinct F 1s peak appears at 684.6 eV, which is absent in the pristine CCFs, providing direct evidence of successful fluoride uptake.

The N 1s spectrum of pristine CCFs consists of two components at 399.2 eV and 402.5 eV, corresponding to amino groups (–NH–/–NH_2_) and quaternary ammonium groups (R_4_N^+^), respectively. Following fluoride adsorption, the binding energy of the amino-related peak remains essentially unchanged, whereas the R_4_N^+^ peak shifts slightly from 402.5 to 402.6 eV, accompanied by a decrease in peak intensity. This change indicates that quaternary ammonium groups are directly involved in fluoride adsorption, while the participation of amino groups is not clearly supported by XPS evidence. The Cl 2p spectrum of pristine CCFs displays two peaks at 197.5 and 199.1 eV, corresponding to Cl 2p_3/2_ and Cl 2p_1/2_ of chloride ions associated with R_4_N^+^ groups [19]. After fluoride adsorption, the binding energies remain unchanged, but a pronounced decrease in peak intensity is observed, suggesting that fluoride adsorption proceeds via an anion exchange process between fluoride and chloride ions.

#### 2.2.5. Calculation of Ion Exchange Between F^−^ and Cl^−^

To further verify the anion exchange mechanism, DFT calculations were conducted to compare the electrostatic adsorption energies of fluoride and chloride ions on the R_4_N^+^ groups of CCFs (Figure S3). The adsorption energy of fluoride (−260.73 kJ/mol) is substantially more negative than that of chloride (−143.99 kJ/mol), indicating that replacement of chloride by fluoride on quaternary ammonium sites is thermodynamically favorable.

Experimental quantification of ion exchange further supports this conclusion. When the fluoride adsorption capacity reached 25.3 mg/g, the fluoride concentration in solution decreased by 1.33 mmol/L, while the chloride concentration increased by 1.37 mmol/L. In contrast, control experiments conducted in deionized water under identical conditions showed a background chloride release of only 0.04 mmol/L. After correction for this background release, the net increase in chloride concentration closely matches the decrease in fluoride concentration, both amounting to 1.33 mmol/L. This near-stoichiometric relationship provides compelling quantitative evidence that fluoride adsorption by CCFs is governed by anion exchange with chloride ions.

#### 2.2.6. Summary of Adsorption Mechanism

In summary, DFT calculations suggest that hydrogen bonding between fluoride ions and hydroxyl or amino groups on chitosan is thermodynamically feasible under idealized conditions. FTIR spectra reveal minor perturbations of these functional groups following fluoride adsorption, indicating possible secondary interactions. However, pristine chitosan fibers exhibit an extremely low fluoride adsorption capacity (1.6 mg/g), demonstrating that hydrogen bonding alone is insufficient to drive effective fluoride removal in aqueous systems.

In contrast, quaternary ammonium-functionalized CCFs exhibit a 15.8-fold increase in fluoride adsorption capacity, accompanied by clear XPS evidence of R_4_N^+^ involvement and quantitative stoichiometric correlation between fluoride uptake and chloride release. These results demonstrate that electrostatic attraction between fluoride ions and quaternary ammonium groups, followed by anion exchange with chloride ions, constitutes the dominant mechanism governing fluoride adsorption on CCFs. Hydrogen bonding interactions likely play a secondary or auxiliary role.

## 3. Conclusions

In this study, quaternary ammonium-functionalized cationic chitosan fibers (CCFs) were successfully developed and demonstrated to be highly efficient adsorbents for the removal of fluoride from aqueous systems, particularly under low-concentration conditions. The adsorption of fluoride onto CCFs proceeds rapidly, reaching equilibrium within 10 min, and the process is well described by the pseudo-second-order kinetic model. Adsorption isotherm analysis based on the Langmuir model yields a maximum fluoride adsorption capacity of 28.5 mg/g under near-neutral pH conditions. CCFs exhibit excellent regeneration performance and cyclic stability. Efficient fluoride desorption can be achieved within 3 min using a mild NaCl solution (0.02 mol/L), and the adsorption performance remained stable after five consecutive adsorption–desorption cycles. These features highlight the practical feasibility and economic attractiveness of CCFs for repeated use in water treatment applications. The practical applicability of CCFs was further validated using five natural surface water samples collected from lakes and rivers. Despite the presence of competing ions and natural organic matter, increasing the CCFs dosage to 0.25 g per 10 mL reduced the effluent fluoride concentration below the WHO drinking water guideline (<1.5 mg/L), while a dosage of 0.4 g achieved fluoride levels below 1.0 mg/L, meeting the Chinese drinking water standard. These results demonstrate the robustness of CCFs under complex water matrices.

Mechanistic investigations combining DFT calculations, FTIR spectroscopy, XPS analysis, and quantitative ion balance experiments reveal that fluoride adsorption is dominated by electrostatic attraction between fluoride ions and quaternary ammonium groups (R_4_N^+^) on the CCFs surface, accompanied by anion exchange with chloride ions. Although hydrogen bonding interactions with hydroxyl or amino groups may occur, they play a secondary role in the overall adsorption process. The introduction of quaternary ammonium functionalities is therefore the key factor responsible for the remarkable enhancement in fluoride adsorption performance. Overall, the CCFs developed in this study integrate high adsorption efficiency, ultrafast kinetics, excellent regenerability, and a metal-free, environmentally benign composition. These advantages make CCFs a promising bio-based adsorbent for advanced purification of low-concentration fluoride-containing water, with potential applications in drinking water treatment, industrial wastewater polishing, and groundwater remediation. Future research can involve packing CCFs into adsorption columns to systematically assess their performance, stability, and regeneration in dynamic continuous flow, yielding key design parameters and process data for practical water treatment applications.

## 4. Materials and Methods

### 4.1. Materials

Chitosan, with a viscosity of 200–220 cP at 20 °C and a deacetylation degree of 85.9%, was obtained from YBBio Co., Ltd. (Yeongdeok, Republic of Korea). Epichlorohydrin (ECH, ≥99.5%), acetic acid (≥99.7%), hydrochloric acid (~36 wt.%), sodium hydroxide (≥98%), (3-chloro-2-hydroxypropyl)trimethylammonium chloride solution (CHTAC, 60 wt.% in water), sodium fluoride (≥98%), sodium bicarbonate (≥99.5%), sodium phosphate monobasic dihydrate (≥99.6%), sodium sulfate (≥99.0%), sodium chloride (≥99.5%) and sodium nitrate (≥99.5%) were all purchased from Sinopharm Chemical Reagent Co., Ltd. (Shanghai, China). Humic acid (≥99%) was got from Shanghai Aladdin Biochemical Technology Co., Ltd. (Shanghai, China). All other chemicals used were analytical grade and were used without further purification.

### 4.2. Preparation of Cationic Chitosan Fibers

Cationic chitosan fibers (CCFs) were prepared following the same process as described in our previous article [19]. Briefly, chitosan fibers (CFs) were prepared via the wet spinning method using 1.0 mol/L NaOH solution as the coagulation bath. To enhance the chemical stability and introduce functional groups, the obtained CFs were cross-linked with epichlorohydrin (ECH) solution at pH 11, followed by reaction with CHTAC solution, resulting in cationic chitosan fibers (CCFs). The product was thoroughly rinsed with deionized water and freeze-dried at approximately −40 °C for subsequent use.

### 4.3. Adsorption Experiments

The fluoride stock solution (200 mg/L) was prepared by dissolving 0.2 g of sodium fluoride in 1000 mL of deionized water and then diluted to the required concentrations for subsequent experiments. Batch adsorption was conducted at 25 °C in 50 mL polyethylene centrifuge tubes containing 0.01 g CCFs and 10 mL of fluoride solution. To investigate the effect of initial pH on fluoride adsorption, the pH of the fluoride solution was adjusted from 1.5 to 10.0 before adding CCFs. The mixtures were then shaken at 150 rpm for 2 h in a temperature-controlled shaker at 25 °C. Adsorption kinetics and isotherm experiments were performed using fluoride solutions at their inherent pH (unadjusted, near-neutral). The reusability of CCFs was evaluated using NaOH, HCl, and NaCl solutions as desorbing agents. The fluoride removal rate (*R*, %), adsorption capacity (*q*, mg/g), and desorption efficiency (%) were calculated using Equations (9)–(11), respectively:

(9) $$R=\frac{\left(c_{i}-c_{f}\right)\times 100}{c_{i}}$$

(10) $$q=\frac{\left(c_{i}-c_{f}\right)\times v}{m}$$

(11) $$\mathrm{Desorption}\;\mathrm{efficiency}\;\left(\%\right)=\frac{\mathrm{Amount}\;\mathrm{of}\;F^{-}\;\mathrm{desorbed}}{\mathrm{Amount}\;\mathrm{of}\;F^{-}\;\mathrm{adsorbed}}\;\times \;100$$

where *c_i_* and *c_f_* are the fluoride ion concentrations (mg/L) before and after adsorption, respectively. v is the adsorption liquid volume (L); *m* is the amount of adsorbent (g).

### 4.4. DFT Calculation Method

Quantum chemistry calculations were performed to elucidate the atomic-level fluoride adsorption mechanism on CCFs, employing the DMol^3^ module in Materials Studio (Version 2020, Accelrys Software Inc., San Diego, CA, USA). Electronic structures were obtained by solving the Kohn–Sham equation under self-consistently unrestricted spin conditions. Exchange–correlation interactions were modeled using the Perdew–Burke–Ernzerh generalized gradient approximation, incorporating DFT Semi-core pseudopods and double numerical polarization functions. Van der Waals forces were accounted for via the DFT +D method (Tkatchenko–Scheffler scheme). Convergence was accelerated by a Fermi smearing of 0.005 Ha [25,45]. Adsorption energy (*E_ads_*, kJ/mol) was determined according to Equation (12).

(12) $$E_{ads}=(E_{F^{-}\mathrm{or}{\mathrm{Cl}}^{-}\mathrm{loaded}\;\mathrm{CCFs}}-E_{F^{-}\mathrm{or}{\mathrm{Cl}}^{-}}-E_{\mathrm{CCFs}})\times 2625.5$$

where $E_{F^{-}\mathrm{or}{\mathrm{Cl}}^{-}\mathrm{loaded}\;\mathrm{CCFs}}$ is the total energy after equilibrium (Ha); $E_{F^{-}\mathrm{or}{\mathrm{Cl}}^{-}}$ and *E*_CCFs_ represent the energies of F^−^ or Cl^−^ and CCFs, respectively (Ha).

### 4.5. Adsorbents Characterization

Fluoride concentration was determined using ion chromatography (ICS-6000, Thermo Fisher, Waltham, MA, USA). Changes in CCFs surface functional groups, pre- and post-adsorption, were characterized by Fourier transform infrared spectroscopy (FT-IR, Nicolet iS5, Thermo Fisher, USA) using the attenuated total reflection (ATR) technique over the 400–4000 cm^−1^ range. Chemical state changes on the surface of CCFs before and after fluoride adsorption were recorded by X-ray Photoelectron Spectroscopy (XPS, Nexsa, Thermo, Waltham, MA, USA). Additionally, the XPS test data were analyzed using XPSPEAK41 software based on the C–C/C–H characteristic peak at 284.8 eV.

## Figures and Tables

**Table 1:** Parameters of kinetics and isotherms.

Models		Parameters	Values
Kinetics		*q_e_* (mg/g)	20.33 ± 0.92
	Pseudo-first-order	*k*_1_ (L/min)	2.09 ± 0.32
		*q*_1_ (mg/g)	19.12 ± 0.52
		*R* ^2^	0.95
	Pseudo-second-order	*k*_2_ (g/mg·min)	0.18 ± 0.03
		*q*_2_ (mg/g)	20.24 ± 0.53
		*R* ^2^	0.97
Isotherm	Langmuir	*b* (L/mg)	0.024 ± 0.003
		*q_max_* (mg/g)	28.5 ± 1.4
		*R* ^2^	0.98
		χ^2^_red_	0.92
	Freundlich	*k_F_* ((mg/g)(L/mg)^1/*n*^)	2.44 ± 0.29
		*n*	2.23 ± 0.13
		*R* ^2^	0.98
		χ^2^_red_	1.04
Thermodynamic	Δ*G*° (kJ/mol)	25 °C	−13.4
		35 °C	−14.3
		45 °C	−15.3
	Δ*H*° (kJ/mol)		15.0
	Δ*S*° (kJ/mol·K)		0.095

**Table 2:** The fluoride adsorption performance of various chitosan-based adsorbents.

Adsorbents	T (°C)	pH	*q_m_* (mg/g)	Equilibrium Time (min)	*k*_1_ (L/min)	*k*_2_ (g/mg·min)	Ref.
Chitosan/FeOOH/AlOOH beads	35	3.5	71.9	300	0.01	0.01	[27]
Zirconium/lanthanum modified chitosan/polyvinyl alcohol	25	7	30.7	15	0.02	2.32	[26]
Lanthanum/chitosan/polyvinyl alcohol	25	7	28.7	15	0.022	1.27	[26]
chitosan/aluminum hydroxide bead	25	/	23.1	90	0.04	˂0.01	[28]
Zirconium modified chitosan/polyvinyl alcohol	25	7	20.8	15	0.02	0.07	[26]
Chitosan@Zirconium	25	7.0	17.0	60	0.04	0.02	[29]
chitosan-praseodymium complex	27	7	15.9	60	0.02	0.01	[30]
La(III) incorporated carboxylated chitosan	30	neutral	11.99	40	0.18	0.01	[31]
zirconium (IV)-magnetic chitosan GO	25	4	8.7	180	0.17	0.04	[32]
La(III)-loaded bentonite/chitosan beads	30	5	8.5	480	/	˂0.01	[33]
Eco-magnetic nano-hydroxyapatite chitosan	30	neutral	8.2	30	/	/	[22]
Protonated cross-linked chitosan particles	/	7	8.1	40	/	/	[34]
Zr(IV) impregnated dithiocarbamate chitosan	/	7	7.3	40	0.15	0.02	[35]
Chitosan supported La(III) and Zr(IV)	30	neutral	6.4	40	/	/	[36]
Ionic liquid-functionalized chitosan	25	4	5.9	/	/	/	[37]
Nano-hydroxyapatite/chitosan	30	neutral	2.0	30	0.21	0.15	[38]
Hydrotalcite/chitosan composite	30	neutral	1.9	30	0.09	0.19	[39]
Chitosan encapsulated tricalcium phosphate	30	/	1.6	30	0.12	0.17	[40]
Aluminum based chitosan/zeolite molecular sieve composite	20	7	1.3	480	0.01	0.16	[41]
Cationic Chitosan Fibers	25	~7	28.5	10	2.09	0.18	This study
	25	3.5	66.6	/	/	/	

**Table 3:** Anion and organic matter concentrations in the surface water samples.

Sample	Longitude	Latitude	F^−^	Cl^−^	NO_3_^−^	PO_4_^3−^	SO_4_^2−^	HCO_3_^−^	TP	TOC
East Lake	114.3605	30.5549	0.304	28.4	0.164	ND	36.3	195	0.13	9.19
Han River	114.2588	30.5720	0.192	10.7	1.260	ND	25.4	146	0.09	4.92
Liangzi Lake	114.4659	30.2514	0.242	8.33	0.342	ND	12.9	79	0.05	5.26
South Lake	114.3973	30.4939	0.329	28.8	0.268	0.040	34.3	110	0.15	8.56
Yangzi River	114.3115	30.5907	0.186	18.4	1.550	ND	32.0	153	0.13	6.67

The concentrations in this table are reported in mg/L; ND indicates not detected.

## Data Availability

The original contributions presented in this study are included in the article/Supplementary Material.

## Supplementary Materials

The following supporting information can be downloaded at: https://www.mdpi.com/article/10.3390/gels12030195/s1, Figure S1: Adsorption of fluoride ions by CFs and CCFs; Figure S2: SEM images of CCFs: (a,b) before F-loading (×1500, ×100), and (c,d) after F-loading (×1500, ×100); Figure S3: DFT calculated adsorption energies (Eads) of F^−^ and Cl^−^ on CCFs; Table S1: FT-IR peak assignments for CCFs and F-loaded CCFs.

## References

[1] Zhao Z., Mi Y., Wang S., Du X., Zhang Q. (2025). The high pKa-guided defect engineering: Improving fluoride removal in actual scenarios by benzimidazole modulated metal-organic frameworks. Water Res..

[2] Hu W., Tang Y., Wang K., Xie F., Wu C., Li W., Chen C., Huang X., Wang J., Ye C. (2025). Rapid and deep removal of fluorine from wastewater by porous La-Al layered double hydroxides material: Reaction mechanism coupled with chemical precipitation and adsorption. J. Water Process Eng..

[3] Patrick M., Sahu O. (2023). Origins, Mechanisms, and Remedies of Fluoride Ions from Ground and Surface Water: A Review. Chem. Afr..

[4] Kumar P., Kumar M., Barnawi A.B., Maurya P., Singh S., Shah D., Yadav V.K., Kumar A., Kumar R., Yadav K.K. (2024). A review on fluoride contamination in groundwater and human health implications and its remediation: A sustainable approaches. Environ. Toxicol. Pharmacol..

[5] Ho H.-J., Takahashi M., Iizuka A. (2023). Simultaneous removal of fluoride and phosphate from semiconductor wastewater via chemical precipitation of calcium fluoride and hydroxyapatite using byproduct of recycled aggregate. Chemosphere.

[6] Zhang N., Yang Y., Fan L., Zheng X., Wang J., Jiang C., Xu S., Xu H., Wang D. (2023). Coagulation effect of polyaluminum-titanium chloride coagulant and the effect of floc aging in fluoride removal: A mechanism analysis. Sep. Purif. Technol..

[7] Shen J., Schäfer A. (2014). Removal of fluoride and uranium by nanofiltration and reverse osmosis: A review. Chemosphere.

[8] Singh S., German M., Chaudhari S., Sengupta A.K. (2020). Fluoride removal from groundwater using Zirconium Impregnated Anion Exchange Resin. J. Environ. Manag..

[9] Wang Z., Su J., Zhao T., Li J., Zhang L. (2024). Enhanced removal of fluoride from groundwater using biosynthetic hydroxyapatite modified by bimetallic (La–Fe or La–Al) hydroxides. J. Clean. Prod..

[10] Lacson C.F.Z., Lu M.-C., Huang Y.-H. (2021). Chemical precipitation at extreme fluoride concentration and potential recovery of CaF_2_ particles by fluidized-bed homogenous crystallization process. Chem. Eng. J..

[11] Zhang Y., Wang L., Zhang R., He C., Jia L., Wang X., Feng X., Jiang T., Xie B., Ma X. (2023). Steering Electron Density of Zr Sites Using Ligand Effect in Bio-Beads for Efficient Defluoridation. Adv. Funct. Mater..

[12] Zhao M.M., Wang Q., Krua L.S.N., Yi R.N., Zou R.J., Li X.Y., Huang P. (2023). Application Progress of New Adsorption Materials for Removing Fluorine from Water. Water.

[13] Liu D., Li Y., Liu C., Li B. (2023). Porous Lanthanum-Zirconium phosphate with superior adsorption capability of fluorine for water treatment. J. Colloid. Interface Sci..

[14] Chen J., Yang R., Zhang Z., Wu D. (2022). Removal of fluoride from water using aluminum hydroxide-loaded zeolite synthesized from coal fly ash. J. Hazard. Mater..

[15] Devi B., Baruah N.P., Bharadwaj A., Devi A. (2023). Adsorptive removal of fluoride ions from aqueous solution using activated carbon supported tetrametallic oxide system. Chem. Eng. Res. Des..

[16] Alhassan S.I., Wang H., He Y., Yan L., Jiang Y., Wu B., Wang T., Gang H., Huang L., Jin L. (2022). Fluoride remediation from on-site wastewater using optimized bauxite nanocomposite (Bx-Ce-La@500): Synthesis maximization, and mechanism of F^─^ removal. J. Hazard. Mater..

[17] Liu F., Wang Q., Li Y., Zhou Z., Wang N., Wang T., Huang X., Hao H. (2025). 3D crosslinked chitosan for fluoride remediation in industrial wastewater: From structure to performance enhancement. Sep. Purif. Technol..

[18] Lin X., Song M.-H., Lei L., Tran D.T., Shu Y., Lim C.-R., Wu X., Mao J., Yun Y.-S. (2025). Rapid and efficient recovery of Au(I) from cyanide gold leachate via quaternary ammonium-functionalized chitosan fibers: Insights into synthesis mechanism and adsorption behavior. Sep. Purif. Technol..

[19] Lin X., Song M.-H., Li W., Wei W., Wu X., Mao J., Yun Y.-S. (2024). Optimized design of quaternary amino-functionalized chitosan fibers for ultra-high diclofenac adsorption from wastewater. Chemosphere.

[20] Zhang Y., Guo Z., Liu P., Qiu Z., Gitis V., Feng H., Li Y., Cai Y., Xiang H., Li H. (2024). Hydrogen-bond dominated phosphorus uptake by chitosan-calcium alginate coated melamine foam in ecological floating beds. Chem. Eng. J..

[21] Pandi K., Viswanathan N. (2015). Synthesis and applications of eco-magnetic nano-hydroxyapatite chitosan composite for enhanced fluoride sorption. Carbohydr. Polym..

[22] Muththamizh B., Sowmya A., Rajesh M. (2025). Zirconium ion incorporated beta-cyclodextrin and chitosan composites for nitrate and fluoride pollution remedy. Int. J. Biol. Macromol..

[23] Hu R., Tian Z., Fan Z., Huang T., Wen G. (2024). Pilot test on acidic fluoride-containing wastewater treatment in the photovoltaic industry through induced crystallization with a focus on calcium fluoride recovery. Sep. Purif. Technol..

[24] Kandel D.R., Poudel M.B., Radoor S., Chang S., Lee J. (2024). Decoration of dandelion-like manganese-doped iron oxide microflowers on plasma-treated biochar for alleviation of heavy metal pollution in water. Chemosphere.

[25] Lin X., Xu Y., Tian Z., Wu G., Wu X., Zan F., Lu X., Cui L., Mao J. (2026). Quantitative mechanistic understanding of Sn(II) recovery using metal-free crosslinked alginate fibers. Chem. Eng. J..

[26] Mei L., Wei J., Yang R., Ke F., Peng C., Hou R., Liu J., Wan X., Cai H. (2023). Zirconium/lanthanum-modified chitosan/polyvinyl alcohol composite adsorbent for rapid removal of fluoride. Int. J. Biol. Macromol..

[27] Liu R., Xu W., Zhou L., Ye L., Luo X., Fan S. (2025). Honeycomb-inspired chitosan-based beads with pore structure and multifunctional groups: Improvement of fluoride ion adsorption efficiency and DFT calculations. Int. J. Biol. Macromol..

[28] Hu H., Yang L., Lin Z., Zhao Y., Jiang X., Hou L. (2018). A low-cost and environment friendly chitosan/aluminum hydroxide bead adsorbent for fluoride removal from aqueous solutions. Iran. Polym. J..

[29] (2025). Mechanistic Study of Fluoride Removal from Aqueous Media Using Chemically Modified Chitosan@Zirconium Adsorbent. Int. J. Thin Film. Sci. Technol..

[30] Kusrini E., Sofyan N., Suwartha N., Yesya G., Priadi C.R. (2015). Chitosan-praseodymium complex for adsorption of fluoride ions from water. J. Rare Earths.

[31] Viswanathan N., Meenakshi S. (2008). Enhanced fluoride sorption using La(III) incorporated carboxylated chitosan beads. J. Colloid. Interface Sci..

[32] Liu M., Zang Z., Zhang S., Ouyang G., Han R. (2021). Enhanced fluoride adsorption from aqueous solution by zirconium (IV)-impregnated magnetic chitosan graphene oxide. Int. J. Biol. Macromol..

[33] Zhang Y., Xu Y., Cui H., Liu B., Gao X., Wang D., Liang P. (2014). La(III)-loaded bentonite/chitosan beads for defluoridation from aqueous solution. J. Rare Earths.

[34] Huang R., Yang B., Liu Q., Ding K. (2012). Removal of fluoride ions from aqueous solutions using protonated cross-linked chitosan particles. J. Fluor. Chem..

[35] Liu B., Wang D., Yu G., Meng X. (2013). Removal of F^−^ from aqueous solution using Zr(IV) impregnated dithiocarbamate modified chitosan beads. Chem. Eng. J..

[36] Muthu Prabhu S., Meenakshi S. (2014). Enriched fluoride sorption using chitosan supported mixed metal oxides beads: Synthesis, characterization and mechanism. J. Water Process Eng..

[37] Dzieniszewska A., Nowicki J., Rzepa G., Kyziol-Komosinska J., Semeniuk I., Kiełkiewicz D., Czupioł J. (2022). Adsorptive removal of fluoride using ionic liquid-functionalized chitosan—Equilibrium and mechanism studies. Int. J. Biol. Macromol..

[38] Sairam Sundaram C., Viswanathan N., Meenakshi S. (2008). Uptake of fluoride by nano-hydroxyapatite/chitosan, a bioinorganic composite. Bioresour. Technol..

[39] Viswanathan N., Meenakshi S. (2010). Selective fluoride adsorption by a hydrotalcite/chitosan composite. Appl. Clay Sci..

[40] Viswanathan N., Kumar I.A., Meenakshi S. (2019). Development of chitosan encapsulated tricalcium phosphate biocomposite for fluoride retention. Int. J. Biol. Macromol..

[41] Gao Y., Liu S. (2022). Adsorption characterization and mechanism of aluminum based chitosan/zeolite molecular sieve composite for fluoride removal. Desalination Water Treat..

[42] Kumar I.A., Jeyaprabha C., Meenakshi S., Viswanathan N. (2019). Hydrothermal encapsulation of lanthanum oxide derived Aegle marmelos admixed chitosan bead system for nitrate and phosphate retention. Int. J. Biol. Macromol..

[43] Zhou Q., Xiang H., Dai Y., Xiong J., Jiang F., Lin Z., Wang S., Yang X. (2026). Rational design of MOF-COF composites for synergistic low-concentration fluoride adsorption: Mechanistic insights and DFT validation. Sep. Purif. Technol..

[44] Zhou D., Zhang L., Guo S. (2005). Mechanisms of lead biosorption on cellulose/chitin beads. Water Res..

[45] Lin X., Shu Y., Dong J., Wu X., Lu X., Zhou T., Mao J., Zan F. (2025). DFT-guided structural design of functionalized chitosan for selective Ag(I) recovery across a broad pH range: Methodology and mechanism. Sep. Purif. Technol..

