# Supplementary material for: Rapid and Efficient Purification of Low-Concentration Fluoride-Containing Water Using Cationic Chitosan Fibers
Source: Gels. 2026 Feb 26;12(3):195. doi: 10.3390/gels12030195 (PMC13025064; doi:10.3390/gels12030195)
Supplement: Supplementary file 1 [file gels-12-00195-s001.zip › gels-4126613-supplementary.pdf]

Rapid and Efficient Purification of Low-Concentration Fluoride-Containing Water Using Cationic Chitosan Fibers

Zhe Liu [1,2,3], Dongfang Wang[2,3], Yan Zhu[2,3], Songlin Wang[1*]

[1]School of Environmental Science and Engineering, Huazhong University of Science and Technology, Wuhan 430074, China；

[2]Hubei Provincial Academy of Eco-Environmental Sciences (Provincial Ecological Environment Engineering Assessment Center), Wuhan 430072, China；

[3]Hubei Key Laboratory of Pollution Damage Assessment and Environmental Health Risk Prevention and Control, Wuhan 430072, China

*Corresponding author email: wangsonglin99@126.com

Table S1. FT-IR peak assignments for CCFs and F-loaded CCFs

groups	CCFs	F-loaded CCFs	Ref.
stretching vibrations of $-NH_2$ and -OH	3276	3271	[1]
the asymmetric stretching vibration of C-H bonds	2922	2919	[2]
stretching vibration and bending vibration of $-NH_2$	1650	1645	[3]
R_4N^+	1477	1477	[4]
-N-H	1373	1373	[2]
C-O-C stretching vibration	1025	1025	[5]
R_4N^+	970	967	[4]

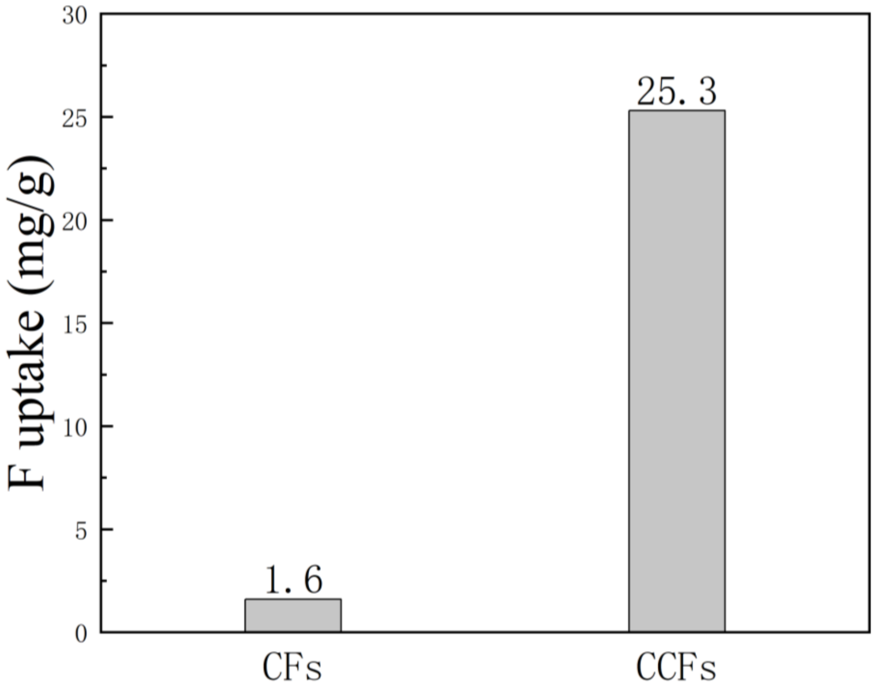

Figure S1 Adsorption of fluoride ions by CFs and CCFs

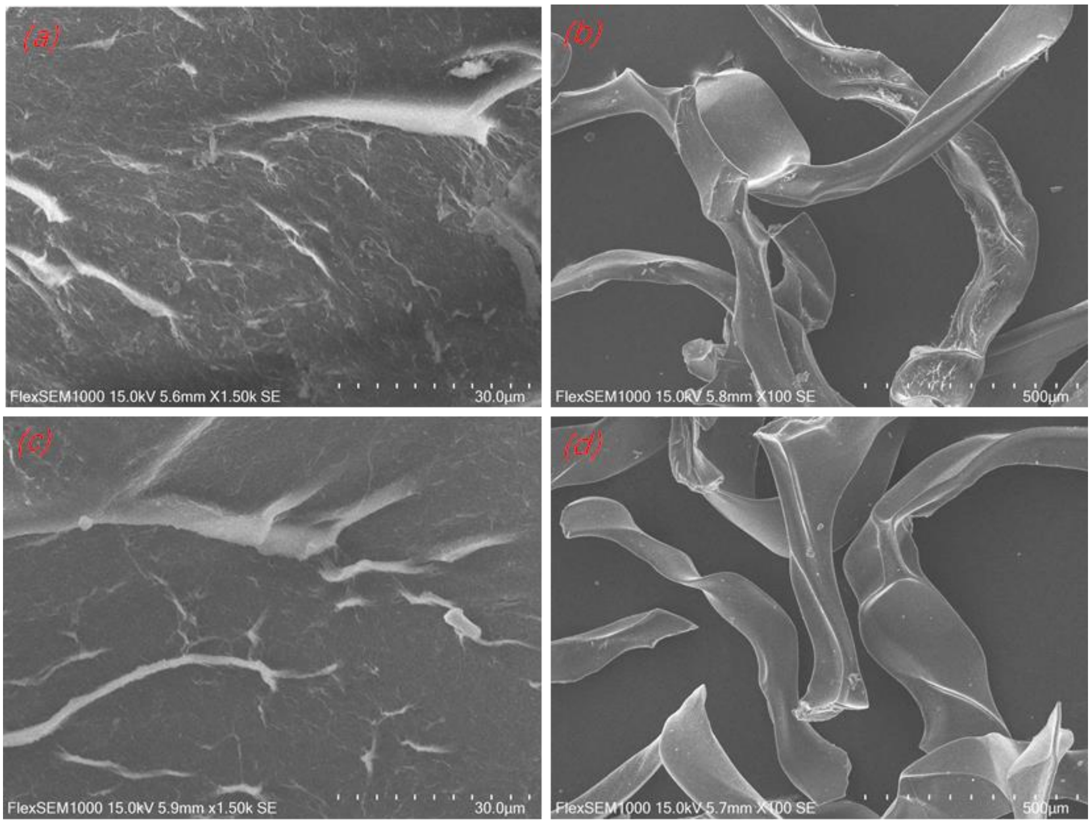

Figure S2. SEM images of CCFs: (a, b) before F- loading (×1500, ×100), and (c, d) after F -loading (×1500, ×100).

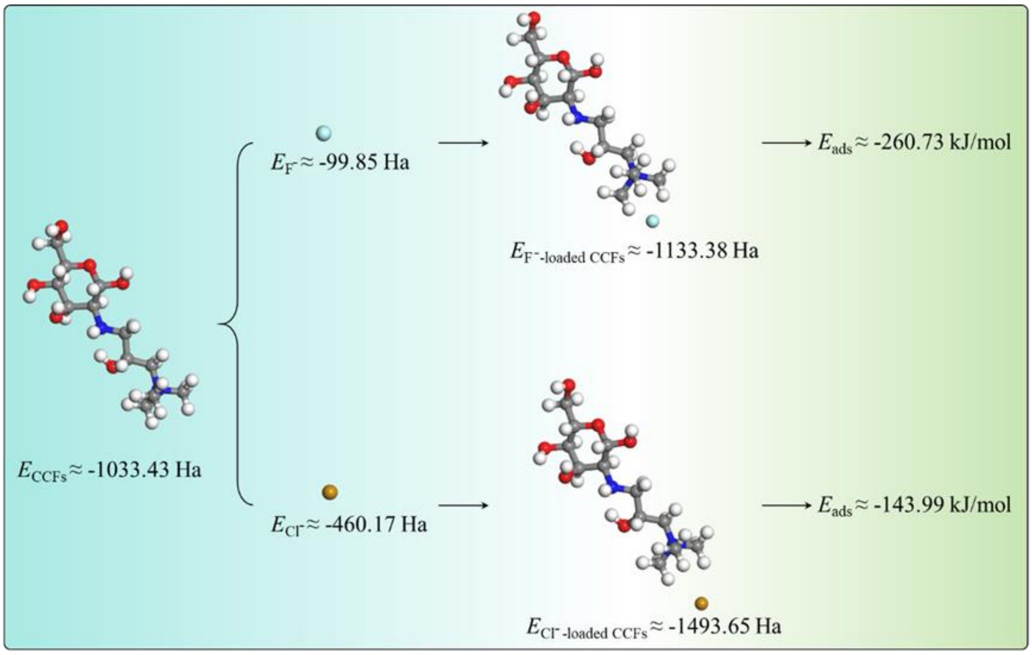

Figure S3 DFT calculated adsorption energies (E_{ads}) of F^- and Cl^- on CCFs.

References

[1] X. Lin, M.-H. Song, L. Lei, D.T. Tran, Y. Shu, C.-R. Lim, X. Wu, J. Mao, Y.-S. Yun, Rapid and efficient recovery of Au(I) from cyanide gold leachate via quaternary ammonium-functionalized chitosan fibers: Insights into synthesis mechanism and adsorption behavior, Separation and Purification Technology, 362 (2025).

[2] I.A. Kumar, C. Jeyaprabha, S. Meenakshi, N. Viswanathan, Hydrothermal encapsulation of lanthanum oxide derived Aegle marmelos admixed chitosan bead system for nitrate and phosphate retention, International Journal of Biological Macromolecules, 130 (2019) 527-535.

[3] R. Liu, W. Xu, L. Zhou, L. Ye, X. Luo, S. Fan, Honeycomb-inspired chitosan-based beads with pore structure and multifunctional groups: Improvement of fluoride ion adsorption efficiency and DFT calculations, International Journal of Biological Macromolecules, 322 (2025).

[4] X. Lin, M.-H. Song, W. Li, W. Wei, X. Wu, J. Mao, Y.-S. Yun, Optimized design of quaternary amino-functionalized chitosan fibers for ultra-high diclofenac adsorption from wastewater, Chemosphere, 357 (2024).

[5] C. Sairam Sundaram, N. Viswanathan, S. Meenakshi, Uptake of fluoride by nano-hydroxyapatite/chitosan, a bioinorganic composite, Bioresource Technology, 99 (2008) 8226-8230.
